# COVID-19 Disruptions in Health Professional Education: Use of Cognitive Load Theory on Students' Comprehension, Cognitive Load, Engagement, and Motivation

**DOI:** 10.3389/fmed.2021.739238

**Published:** 2021-10-04

**Authors:** Siti Nurma Hanim Hadie, Vina Phei Sean Tan, Norsuhana Omar, Nik Aloesnisa Nik Mohd Alwi, Hooi Lian Lim, Ku Ishak Ku Marsilla

**Affiliations:** ^1^Department of Anatomy, School of Medical Sciences, Universiti Sains Malaysia, Kota Bharu, Malaysia; ^2^School of Health Sciences, Universiti Sains Malaysia, Kota Bharu, Malaysia; ^3^Department of Physiology, School of Medical Sciences, Universiti Sains Malaysia, Kota Bharu, Malaysia; ^4^School of Dental Sciences, Universiti Sains Malaysia, Kota Bharu, Malaysia; ^5^School of Educational Studies, Universiti Sains Malaysia, Penang, Malaysia; ^6^School of Materials and Mineral Resources Engineering, Universiti Sains Malaysia, Nibong Tebal, Malaysia

**Keywords:** cognitive load theory, cognitive load, motivation, engagement, online lecture, lecture comprehension

## Abstract

This study explored the impact of online lectures that were developed using principles of cognitive load theory (CLT) and cognitive theory of multimedia learning (CTML) on health profession students' lecture comprehension, cognitive load, cognitive engagement, and intrinsic motivation in learning. A total of 215 first-year undergraduate students in medical, dentistry, and nutrition programs participated in this pre-post quasi experimental study. The students attended a typical face-to-face lecture on Day-1 of the intervention, followed by a CLT-based online lecture 8 weeks thereafter. Their comprehension of the lecture topics was measured through pre- and post-lecture assessments, and their cognitive load, cognitive engagement, and motivation were measured immediately after each lecture session. The analysis revealed that the CLT-based online lectures promoted the students' comprehension of the lecture content (*p* < 0.001), self-perceived learning (*p* < 0.001), engagement toward the learning material, and motivation to learn (*p* = 0.025). It was also effective at reducing the students' intrinsic and extraneous cognitive loads (*p* < 0.001). Hence, designing online lectures using CLT and CTML principles could be an effective method to promote students' knowledge and comprehension, cognitive engagement, and learning motivation. However, further research is needed to investigate the applicability and impact of CLT-based online lectures in non-health profession disciplines.

## Introduction

Lecturing is commonly regarded as a teaching method that involves information transfer from a teacher to a group of learners (1). Traditional didactic lectures are often viewed as a “sage on the stage method” because they are teacher- centered, and students are passive recipients of information (2). As such, the traditional didactic lecture approach has long been criticized for its inability to stimulate deep learning and knowledge acquisition (3–6). Despite these criticisms, a review of the roles of lectures in higher education revealed that lectures are still an important and relevant teaching method, particularly because they offer meaningful benefits, including pedagogical (i.e., content framework of a subject), practical (i.e., listening and notetaking skills), and social (i.e., cost effective and shared communal understanding) in nature (7). In fact, modern lectures have evolved beyond the capacity of the traditional lecture and are commonly delivered in a more engaging and interactive way (8, 9). Several published guidelines on lecturing suggest that lectures can be more effective when practiced alongside other teaching modalities and when the delivery adopts educational principles (1, 10–12). With these innovations, the practice of lecturing has become more flexible in catering to diverse student learning styles and changes in the learning environment.

The SARS-CoV-2 (COVID-19) pandemic has changed our lecturing environment, causing a shift from face-to-face to online lectures (13). Prior to the COVID-19 pandemic, online lectures in higher education were mainly used in blended and online distance learning and were usually delivered asynchronously as homework or pre-class assignments (14, 15). In other words, online lectures were not comprehensively practiced by all faculty members. However, the COVID-19 pandemic has resulted in an abrupt suspension of face-to-face lectures and the exclusive adoption of online lectures by faculty members in many higher education institutions. In fact, recent descriptions of online lectures during COVID-19 suggest that lecture design and delivery have diversified beyond the routinely practiced approach (16). Online lectures are typically delivered synchronously through online platforms (i.e., Webex, Zoom, Google Classroom, and Microsoft Teams) that promote interactive conferences and live discussion as well as asynchronously through the university's learning management system in order to cater to the learning needs of students who have limited access to high-speed Internet connections (17). Nevertheless, this abrupt transition to online lectures has presented significant challenges for faculty members as they have had to rapidly develop their digital competency skills for adoption in their teaching (18). In addition, students have highlighted several barriers to online learning, even before the COVID-19 pandemic, as they often perceive online instruction and delivery practices to be less effective than blended learning and traditional face-to-face teaching (19). Likewise, a study that explored students' perceptions of online lectures amid COVID-19 through semi-structured interviews revealed that online lectures did not improve students' understanding of subject content due to the lack of interaction, sustained attention focus, and stimulated interest (20). Thus, the design and delivery of online lectures require proper planning to enable meaningful learning experiences. This may be achieved by incorporating instructional design theories that are not usually explicit in the context of online lectures (18).

Online lectures that adopt instructional design theories in their design approach may be necessary to ensure effectiveness in achieving intended learning outcomes. An effective lecture should be able to enhance the development of students' learning competencies, particularly in relation to cognitive, volitional, attitudinal, and behavioral goals (21). In addition, the design of an effective lecture should be more dynamic and should cater to two-way interactions between students and lecturers (22). Consequently, an effective online lecture should be able to stimulate students' cognitive engagement and motivation and reduce their cognitive burden through interaction and intra-lecture activities. This could be accomplished by adopting cognitive load theory (CLT) and cognitive theory of multimedia learning (CTML) principles into the design of online lectures (12, 23).

CLT and CTML are instructional design theories that describe optimal learning when instructional material is designed according to the architecture and function of human cognition (24, 25). The core of CLT and CTML principles is working memory, which processes raw information received from sensory memory into meaningful schema, eventually transferring this schema to long-term memory for permanent storage (26). Given that working memory has limited processing and storage capacity, the principles of CLT and CTML have been designed to manage all cognitive input imposed on the working memory system (27). This facilitates information processing and guards against overloading working memory with information. In the CLT context, cognitive input can be categorized into three types: (1) intrinsic load (IL, i.e., input that is relevant to learning) (28), (2) extraneous load (EL, i.e., input that is irrelevant to learning) (29); and (3) germane load (GL, i.e., mental effort used by learners to process relevant learning materials) (29). To achieve optimal learning, the summation of IL and EL—known as the total cognitive load—must not exceed the working memory capacity, and GL should be increased to process learning materials. Based on this paradigm, CLT and CTML outline several empirically proven principles that manage the IL, reduce the EL, and increase the GL of learners when learning complex instructions.

Based on these principles, Hadie et al. (12) developed a lecturing guideline—the CLT-based lecture model—which was studied for its effectiveness in a multi-center randomized controlled trial in several medical anatomy lectures. CLT-based lectures have undergone face-to-face delivery and were found to effectively promote students' knowledge acquisition and retention, self-perceived learning, and cognitive engagement while also reducing medical students' cognitive loads (23). Nevertheless, the potential impact of the CLT-based lecture model on the design and delivery of online lectures has yet to be explored. Indeed, Andersen and Makransky (30) raised concerns over the potential increase in their students' EL, which could have been imposed from noises, media, and devices within the online environment. Research has proven that handling online devices during learning requires students to multitask, thus imposing higher EL (31). Likewise, noise generated from the online environment will cause distraction, thereby hampering learning (32, 33).

The unprecedented COVID-19 pandemic has paved the way to explore the effectiveness of CLT-based online lectures. As the effects of CLT are only observable when applied to complex instruction (26), this study explores the effect of CLT-based online lectures on medical, dental, and allied health professionals. Studies have indicated that topics relating to medical, dental, and allied health professional are among the most complex in higher education as the content is highly integrated with that of other disciplines, covers a wide range of diseases and management, and evolves rapidly over time with advancements in research and technology (34–36). This study explored the impact of CLT-based online lectures across different disciplines on students' learning achievement through comprehension, cognitive load level, cognitive engagement, and motivation. Specifically, we address these research questions—(1) Do CLT-based online lectures enhance comprehension of difficult health professional topics regardless of learning and lecture styles? (2) Do CLT-based applications work in an online setting? and (3) Does CLT-based online teaching provide an advantage to enhance motivation and reduce cognitive loads in learning compared to traditional lectures? We hypothesize that CLT-based online lectures will improve students' lecture comprehension, which is influenced by the improvement of students' engagement and motivation and a reduction in their intrinsic and extraneous load.

**Figure 1:**
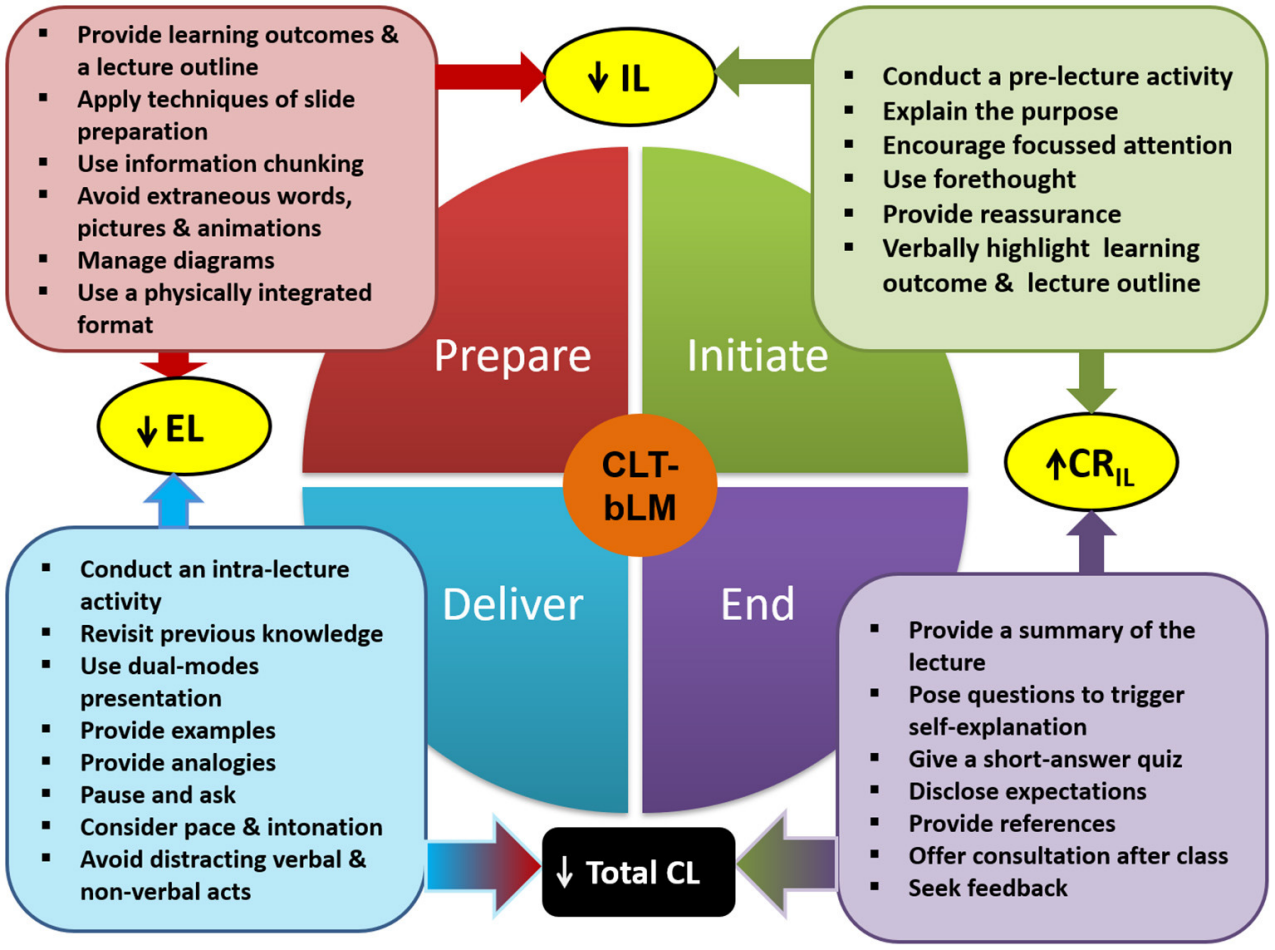
The cognitive load theory-based lecture model [retrieved from (12)].

The CLT-based lecture model is a lecturing guideline developed by Hadie et al. (12) in an attempt to find solutions aimed at increasing the learning of complex anatomy lectures. The model adopts several CLT effects—modality, split-attention, isolated-interacting elements, redundancy, goal-free, guidance fading, worked example, and completion example effects (37)—and CTML principles such as signaling, contiguity, segmenting, coherence, pre-training, personalization, and voice (24). The model contains four phases of lecturing: (1) preparation, (2) initiation, (3) delivery, and (4) end phases, which underlie 27 lecturing strategies [for details, see (12)] (Figure 1).

Briefly, the preparation phase covers the lesson construction and aims to reduce IL and EL by aligning presentations based on clear taxonomy-based learning outcomes. The concept of information chunking is explicitly applied along with guides to manage visual presentation and information load. In the initiation phase, students' prior knowledge is stimulated in order to connect their readiness to learn the topic and focus attention (38), thus increasing their GL before the topic content is presented. During the delivery phase, the focus is to reduce EL by having lecturers revisit previous knowledge, use of proper lecture pacing and voice, and the avoidance of distracting verbal and non-verbal acts. The last phase or end phase aims to increase students' cognitive resources to process IL and allow them to assess how much they have learned during the lecture and plan their own self-study. This is ensured *via* summaries or short quizzes on lecture content.

In addition, rather than cognitive load, the CTML principles applied in this model emphasize three types of cognitive processing: essential, extraneous, and generative processing (24). Essential processing refers to the cognitive effort invested in processing relevant text and pictorial instruction from a multimedia presentation into sensory and working memory; thus, it involves selecting and organizing the processing task through the segmenting principle (i.e., words and pictures are presented in learner-paced segments) and pre-training principles (i.e., students are exposed to the key elements of instruction prior to class to enhance their understanding of the pictorial materials and text) (39). Extraneous processing—through its contiguity principle (i.e., diagrams and related text are presented together on the same screen and are explained verbally), coherence principle (i.e., extraneous materials are excluded from instructional design and delivery), and signaling principle (i.e., visual and auditory cues are added to highlight important information)—processes unrelated extraneous information, which usually results from poor instructional design (24, 39). Generative processing involves the cognitive effort invested in making sense of acoustic and iconic representations through the personalization (i.e., verbal narration is presented informally) and voice (i.e., narration is spoken by a friendly human voice as opposed to a machine voice) principles (24, 39). Thus, essential, extraneous, and cognitive processing are positively correlated with the manipulation of IL, EL, and GL, respectively (40).

## Materials and Methods

### Study Design and Participant Recruitment

This study adopted the pre-post quasi experimental design. This within-subject study design was selected to control for the differences of students learning styles. A total of 215 undergraduate students from the medical, dentistry, and nutrition programs at Universiti Sains Malaysia voluntarily participated in this study. Prior to their recruitment, the whole batches of the first-year medical and dental and third-year nutrition students were invited to attend a briefing session on the purpose, participation criteria, methodology, and benefits of the study. The students were also briefed on their rights, risk, confidentiality, and method of termination should they opt to withdraw from the study. At the end of the briefing session, written consent was obtained from 215 volunteer participants. All the research activities received ethical approval from the Human Research Ethics Committee, Universiti Sains Malaysia (USM/JEPeM/19070414).

The study was conducted in two phases. The first phase used the typical mode of face-to-face lecture delivery, while the second phase consisted of the CLT-based model training for lecturers to incorporate into their second lesson delivery. Since this was a pre–post intervention, both lectures were attended by the same participants, and within-group changes of the measured outcomes were evaluated. This within-subject study design was selected to control the differences of students learning styles and lecture delivery that are known to influence learning (41, 42). The within-subject research design is considered as a Level 1 quality that is able to provide causal reference (43).

The participants attended the two lecture formats based on their discipline. The lectures were delivered by one volunteer lecturer from each of the medical, dental, and allied health sciences schools. The three lecturers were recruited on the basis that they had at least 5–10 years of experience as lecturers and had no previous exposure to knowledge of CLT- or CTML-based instruction. Since the CLT effect is observable when applied to complex instruction (26), only difficult topics were included in this study. Each lecturer was required to identify two difficult topics within their course that imposed almost the same level of complexity based on the amount of information and interaction. Identification of the topics was conducted using a survey that measures element interactivity effect of CLT (supplementary material 1). The element interactivity principle describes that the number of elements in an instructional material together with their interactions are used to determine the difficulty level of the instruction (26, 37, 44). The three lecturers were requested to rate their opinions using a 5-point Likert scale—(1) Strongly disagree, (2) Disagree, (3) Neither disagree or agree, (4) Agree, and (5) Strongly agree for seven items that measured the element interactivity of their lecture. All lecture topics that were selected in this study had achieved maximum mean difficulty score, which is five (45). Both selected topics were in different modules and were unrelated. Thus, learning the first topic did not expose the students to prior knowledge related to the second topic. The medical topics identified were “principles of electrocardiography” and “thyroid hormones”; the dentistry topics were “nuclei acid metabolism” and “biology of osseointegration”; and the nutrition topics were “medical nutrition therapy for renal failure” and “medical nutrition therapy for HIV and AIDS.”

### Phase 1: Face-to-Face Non-CLT Lecture

The first phase of this study was conducted 1 week prior to the introduction of the CLT-based model to the lecturers. During this phase, the lecturers were asked to prepare a lecture on one of the selected topics based on their routine lecturing practice. Since this phase happened before the COVID-19 pandemic and before the implementation of the national lockdown in Malaysia, the lectures were delivered face-to-face over 1 h in a lecture hall setting. The lectures were also attended by an independent researcher who observed and identified the CLT and CTML strategies that could have been unknowingly fulfilled by the lecturers. This effort aimed to ensure an acceptable difference in terms of the CLT and CTML application between the non-CLT- and CLT-based lectures. Prior to the lecture delivery, the students' baseline knowledge was measured, and post-lecture, their lecture comprehension, cognitive load, cognitive engagement, and motivation were measured.

#### Measurement of Students' Baseline Knowledge and Lecture Comprehension

The participants' baseline knowledge and comprehension of the lecture topic were measured through the pre- and post-lecture assessments, respectively. Since the aim of these assessment was to measure participants' recall and understanding of information, which are equivalent to C1 and C2 level of blooms taxonomy, multiple true-false (MTF) assessment format was applied. Furthermore, MTF format has a greater capability to characterize students' thinking regarding the various response options compared to single true answer in a multiple choice (MC) question structure (46). Pre- and post-lecture assessments contained the same five MTF questions on the lecture topic, but the stem and items for the post-lecture assessment were re-positioned in a different order to avoid pattern memorization effects. The questions were developed based on the allocated learning outcomes (i.e., assessed mixture of recall and applied knowledge) and were vetted by four panels of content experts (e.g., physiologist, dental lecturer, and nutritionist) and one medical educationist. The pre- and post-lecture assessments were distributed in hardcopy format 30 min prior to and immediately after the lecture, respectively. The participants were allocated 30 min to answer all the five questions, by rating each item as either true or false. Each correct answer was given one mark, and no mark was deducted for wrong answer. Percentage of pre- and post-lecture assessment scores were calculated for statistical analysis.

#### Measurement of Students' Cognitive Load

The participants' IL, EL, and GL values were measured immediately following the post-lecture assessment. Using the Cognitive Load Scale, a multidimensional 10-item inventory with a ten-point semantic scale ranging from “not at all the case” to “completely the case” captured cognitive load, with GL considered as student self-perceived learning (SPL) (47). This inventory displayed good construct and internal structure validity in the lecture and problem-based learning contexts (47–50).

#### Measurement of Students' Engagement and Motivation Level

The participants' cognitive engagement and motivation toward learning the topic were measured after the post-lecture assessment. The Learners' Engagement and Motivation Questionnaire, which utilizes a seven-point semantic scale ranging from “not at all true” to “very true” (23), was applied. The inventory contains six items relating to the cognitive engagement domains (Cronbach α = 0.93–0.95) (51) and 12 items on the internal motivation domains (“effort and importance” and “value and usefulness”) of the validated Intrinsic Motivation Inventory developed from the self-determination theory principle (52). The 12 motivation items had good construct validity and adequate reliability (53). The same tools were used in the second lecture.

### Phase 2: CLT-Based Online Lecture

One week after the freestyle lecture delivery, the lecturers—who had no prior knowledge of CLT—were invited to attend a 1-day workshop on CLT-based training. During the workshop, they were introduced to the theory, evidence-based principles, and strategies of the CLT-based online guideline (12). Working examples were provided for each strategy, and the lecturers were given hands-on exposure on how to apply the strategies in their lectures. At the end of the workshop, the lecturers were given a task to prepare a CLT-based online delivery lecture that would be attended by the same participants from the second phase of the study. Eight weeks were allocated for the lecture preparation throughout this period, and the lecturers were able to consult with the researchers when they encountered any issues.

The lectures were designed in four phases—(1) preparation, (2) initiation, (3) delivery, and (4) end phases, as outlined in the CLT-based lecture model (23). During the preparation phase, the lecturers provided a list of learning outcomes and lecture outlines, and then prepared lecture slides with techniques that could reduce students' EL (e.g., to use plain-background slides, headings and sub-headings, visual cues, sans-serif fonts, adequate font size, text treatment, and color coding). The lecturers were also guided on how to perform other cognitive load reduction measures, namely, information chunking of the lecture content, arranging the content into integrated format, managing diagrams that were used in their lectures, and avoiding extraneous elements such as unnecessary labels. In initiation phase, the lecturers were guided on how to begin their lectures effectively by applying techniques that can reduce IL and increase SPL. The lecturers were required to conduct a short pre-lecture activity to stimulate students' prior knowledge and explain the lesson's purpose to encourage students' attention. Lecturers were guided to anticipate future problems in learning; and verbally highlight the learning outcomes and lecture outline at the beginning of the session. During the delivery phase, the lecturers were requested to apply several lecturing strategies that could reduce the EL, namely conducting intra-lecture activities, revisiting previously learned knowledge, applying dual-mode presentation, providing examples and analogies, including a short break after 10–15 min of lecturing, avoiding distracting behaviors, and ensuring appropriate pace and intonation in delivery. During the end phase, the lecturers conducted several lecturing strategies that can enhance students' GL in the form of SPL. This was done by enhancing their intrinsic motivation and helping them to consciously invest their mental effort for information processing. The strategies include summarizing the lecture content, posing questions to trigger self-explanations, providing short quizzes and references, disclosing expectations, and offering consultations after class (23).

The CLT-based lesson was initially planned as a face-to-face session, but in view of the COVID-19 pandemic, lectures were then restricted to online-delivery *via* the Cisco WEBEX teleconferencing application. This occurred ~2 months after the national lockdown came into effect. The shift of study settings from face-to-face to online reflects the reality of the COVID-19 pandemic disruption in higher education. Due to precedents of studies that compared traditional face-to-face lectures to online lectures (54–56), revisions were made to compare “control” lectures as presented in Phase 1 with CLT-based online lectures in Phase 2. The CLT-based online lecture lasted 1 h and was delivered by the same person who delivered the face-to-face non-CLT lecture during the first phase of the study. The lectures were also attended by an independent researcher who observed and assessed the lecture delivery process to ensure that they fulfilled the CLT principles. Furthermore, the students' baseline knowledge was measured before the lecture; using the same inventories, their lecture comprehension, cognitive load, cognitive engagement, and motivation were measured after the lecture.

### Data Analysis

The data were entered into the Statistical Package for the Social Sciences (SPSS) software, version 26 and checked for any data entry errors and missing values. To avoid biased estimates, the missing values were imputed with the observed median for cases of <50% missing value (57). Descriptive and inferential statistics were performed using the SPSS software. Descriptive data analysis was performed to calculate the demographic distribution of the participants. Prior to the statistical analyses, assumptions for each test were checked, and the level of significance (α) was set at 0.05, with a confidence interval of 95%. A paired *t*-test was applied to test the within-group difference of the knowledge test scores as well as the cognitive load, engagement, and motivation scores. In addition, an attempt was made to test the mean difference of the test scores between two types of lectures—freestyle vs. CLT-based online lectures—using independent *t*-test. Multivariable linear regression models were constructed to assess the influence of the participants' engagement, motivation, IL, EL, and SPL on changes in the knowledge scores (post to pre scores) while controlling for gender. Gender was controlled due to the imbalanced ratio of men to women participants. This was done for each phase independently. The overall linear regression equation below depicts the statistical analysis model.


(1) $$\begin{array}{r} y=\;B_{0}+\;B_{1}x_{1}+B_{2}x_{2}+B_{3}x_{3}+...{+B}_{p}x_{p} \end{array}$$

where *y* = change in lecture scores (post–pre)

*B* = unstandardized coefficient related to the *n*-variable.

*x* = related variables correlated with *y* (i.e., engagement, motivation, IL, EL, and SPL).

*B*_0_ = equation constant.

## Results

### Observation of the Non-CLT Face-to-Face and CLT-Based Online Lectures

Through our observations from attending both lectures, the non-CLT face-to-face lectures were noted as having no or minimal visible features of CLT and CTML. The lecturers mainly read from their slides, which were packed with textual materials, and the diagrams used in the lectures had unnecessary labels, which were written in small fonts. Minimal visual cues were used during the non-CLT lectures, and the lecturers did not conduct intra-lecture activities. Furthermore, the CLT-based lecture complied with more than half of the 27 CLT lecture strategies outlined in the aforementioned guideline, which indicated a clear transition between subtopics, well-designed and organized information with the use of headings and subheadings (i.e., information chunking), clear and good-sized diagrams with meaningful labels, an appropriate selection of slide backgrounds, application of visual cues (i.e., color coding and animations), and well-designed intra-lecture activities (i.e., online quizzes using an audio-response system). The lecturers also succeeded in stimulating the learners' prior knowledge through the pre-lecture activity (i.e., short video and quizzes) and provided a meaningful summary at the end of the lecture.

### Student Demographics and Lecture Comprehension

Overall, for one male student, there were three more female students across the three schools (Table 1) and health science students were significantly older than their medical and dental counterparts (*p* < 0.001). The students' understanding of the lecture content was determined through increments in the pre- to post-lecture assessment scores. The analysis revealed that the improvement in the test scores within both the face-to-face freestyle and the CLT-based lectures were highly significant (mean difference ± SEM = 19.5 ± 1.6, *t*-stats = 12.5, df = 214, and *p* < 0.001), indicating that the students' understanding of the lecture content had increased after each lecture. However, the mean difference in the test scores between the two types of lectures was significantly higher in the CLT-based online lecture groups (Table 1), which indicated greater improvements. These findings were consistent across the three disciplines.

**Table 1:** Participant's age and gender, and difference of lecture comprehension scores, motivation, engagement, and cognitive values in mean and standard deviation with statistical differences between Phase 1 non-CLT face-to-face and Phase 2 CLT-based online lectures.

	**Participants**			
	**All (*n* = 215)**	**Medical (*n* = 106)**	**Dental (*n* = 54)**	**Nutrition (*n* = 55)**
Age (year)	21.9 ± 2.1	21.3 ± 2.5	21.2 ± 0.4	23.6 ± 0.8
**Gender**, ***n*** **(%)**				
Male	55 (25.6) 160	37 (34.9) 69	13 (24.1) 41	5 (9.1) 50
Female	(74.4)	(65.1)	(75.9)	(90.9)
**Lecture comprehension (difference of tests scores)**				
Non-CLT face-to-face lecture	34.7 ± 20.9^†^	36.6 ± 20.7^†^	33.6 ± 25.2^†^	32.1 ± 16.5^†^
CLT-based online lecture	54.2 ± 15.	52.6 ± 17.5	59.6 ± 12.2	53.7 ± 11.9
**Motivation**				
Non-CLT face-to-face lecture	5.8 ± 0.7^*^	5.9 ± 0.7	5.8 ± 0.7	5.5 ± 0.6
CLT-based online lecture	5.6 ± 0.7	5.8 ± 0.7	5.6 ± 0.8	5.4 ± 0.6
**Engagement**				
Non-CLT face-to-face lecture	5.1 ± 1.2^†^	5.7 ± 1.0	4.5 ± 1.2^†^	4.7 ± 1.2^†^
CLT-based online lecture	5.7 ± 0.7	5.8 ± 0.7	5.5 ± 0.7	5.5 ± 2.1
**Intrinsic load**				
Non-CLT face-to-face lecture	6.3 ± 2.1^†^	5.9 ± 2.1^†^	7.7 ± 1.6^†^	5.6 ± 2.1^†^
CLT-based online lecture	3.8 ± 2.6	4.5 ± 2.6	3.1 ± 2.5	3.4 ± 2.5
**Extrinsic load**				
Non-CLT face-to-face lecture	2.0 ± 1.8^†^	1.7 ± 1.6^†^	2.3 ± 2.0^†^	2.2 ± 1.8
CLT-based online lecture	0.9 ± 1.7	1.0 ± 1.9	0.7 ± 1.5	0.9 ± 1.1
**Self-perceived learning**				
Non-CLT face-to-face lecture	7.6 ± 1.7^†^	8.1 ± 1.5^†^	7.1 ± 1.6^†^	7.2 ± 1.8
CLT-based lecture	8.5 ± 1.8	8.8 ± 1.6	8.5 ± 1.8	7.8 ± 1.9

* *p < 0.05*,

† *p < 0.001—significant differences between Phase 1 and Phase 2 means*.

### Students' Cognitive Load Level

The students' cognitive loads were significantly reduced for IL (mean difference ± SEM = −2.4 ± 0.22, *t*-stats = −11.2, df = 214, and *p* < 0.001) and EL (mean difference ± SEM = −1.1 ± 0.16, *t*-stats = −6.7, df = 214, and *p* < 0.001), and EL (mean difference ± SEM = −1.1 ± 0.16, *t*-stats = −6.7 , df = 214, and *p* < 0.001) in the CLT-based online lecture compared to the freestyle non-CLT lecture. Conversely, the students' self-perceived learning scores—reflecting GL or accounted as SPL—were found to be significantly increased between the freestyle and CLT-based online lectures. These findings were consistent across the three disciplines, except for the students in nutrition, for whom the differences in EL and SPL were not significant. These results are summarized in Table 1.

### Student Engagement and Motivation Level

In general, the student engagement (mean difference ± SEM = 0.54 ± 0.08, *t*-stats = 6.4, df = 214, *p* < 0.001), and motivation (mean difference±SEM = −0.13 ± 0.06, *t* = −2.3, df = 214, and *p* = 0.03) levels were found to be significantly higher when the students attended the CLT-based online lecture compared to the face-to-face freestyle lecture. Nonetheless, the changes in engagement level in the medical discipline and regarding the motivation level in each discipline were not significant. The results are summarized in Table 1.

### Regression Models

After performing a correlation analysis, the relevant variables were imputed into regression models to understand the influences on the change in the lecture comprehension scores on the typical freestyle and CLT-based lecture for all the participants. In the regression model for the non-CLT lectures (Table 2), the *R*^2^ and adjusted-*R*^2^ values were 0.07 and 0.05, respectively, with a significance value of 0.01 for the model fit. Engagement and SPL appeared to influence changes in the lecture comprehension scores in an inverse (*p* = 0.07) and direct (*p* = 0.07) relationship, respectively.

**Table 2:** Influence of motivation, engagement, intrinsic load, extrinsic load, and self-perceived learning in the change of non-CLT face-to-face lecture scores (post–pre) in all participants (*n* = 215).

	**Unstandardized B**	**SE**	**p-value**	**95% CI**
Motivation	3.97	2.5	0.11	−0.95, 8.9
Engagement	−2.96	1.6	0.07	−6.1, 0.21
Intrinsic load	0.38	0.7	0.58	−0.99, 1.75
Extrinsic load	0.32	1.0	0.75	−1.62, 2.26
Self-perceived learning	2.3	1.3	0.07	−0.15, 4.76
Constant	12.7	14.8	0.39	−16.5, 41.8

For the CLT-based lectures, the regression model consisted of the same variables imputed in the non-CLT lecture regression analysis, with the addition of gender (Table 3). Overall, the *R*^2^ and adjusted-*R*^2^ values were 0.08 and 0.05, respectively, with a significance value of 0.01 for the model fit.

**Table 3:** Influence of gender, motivation, engagement, intrinsic load, extrinsic load, and self-perceived learning in the change of CLT-based online lecture scores (post–pre) in all participants (*n* = 215).

	**Unstandardized B**	**SE**	***p*-value**	**95% CI**
Gender	5.45	2.36	0.02	0.79, 10.1
Motivation	3.16	1.82	0.09	−0.44, 6.77
Engagement	−2.83	1.98	0.16	−6.74, 1.08
Intrinsic load	−0.47	0.42	0.26	−1.30, 0.36
Extrinsic load	−0.09	0.68	0.90	−1.44, 1.26
Self-perceived learning	1.05	0.65	0.11	−0.22, 2.32
Constant	35.94	9.89	<0.001	16.4, 55.4

**Figure 2:**
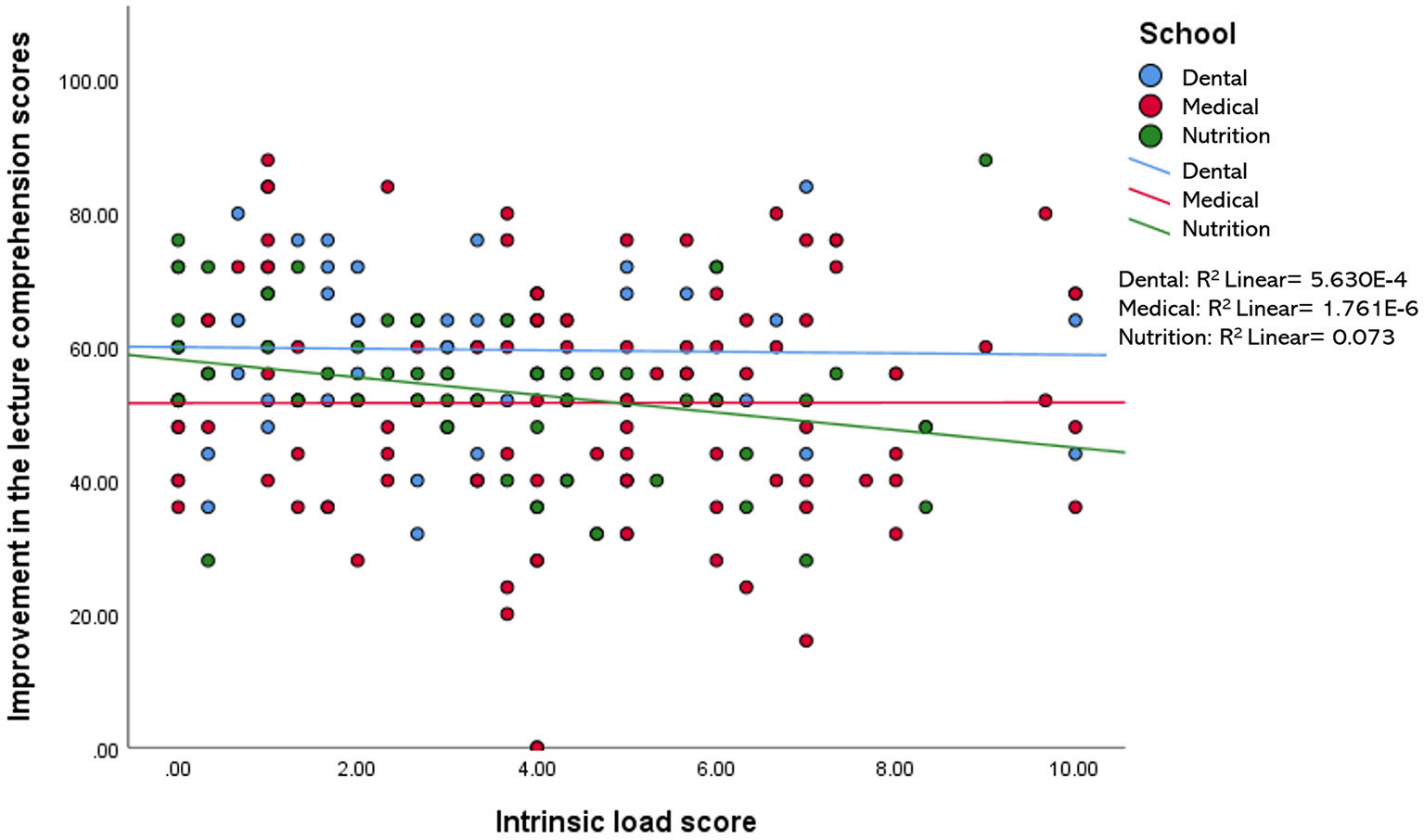
Improvement of the lecture comprehension scores vs. the intrinsic load score after the CLT-based online lecture.

**Figure 3:**
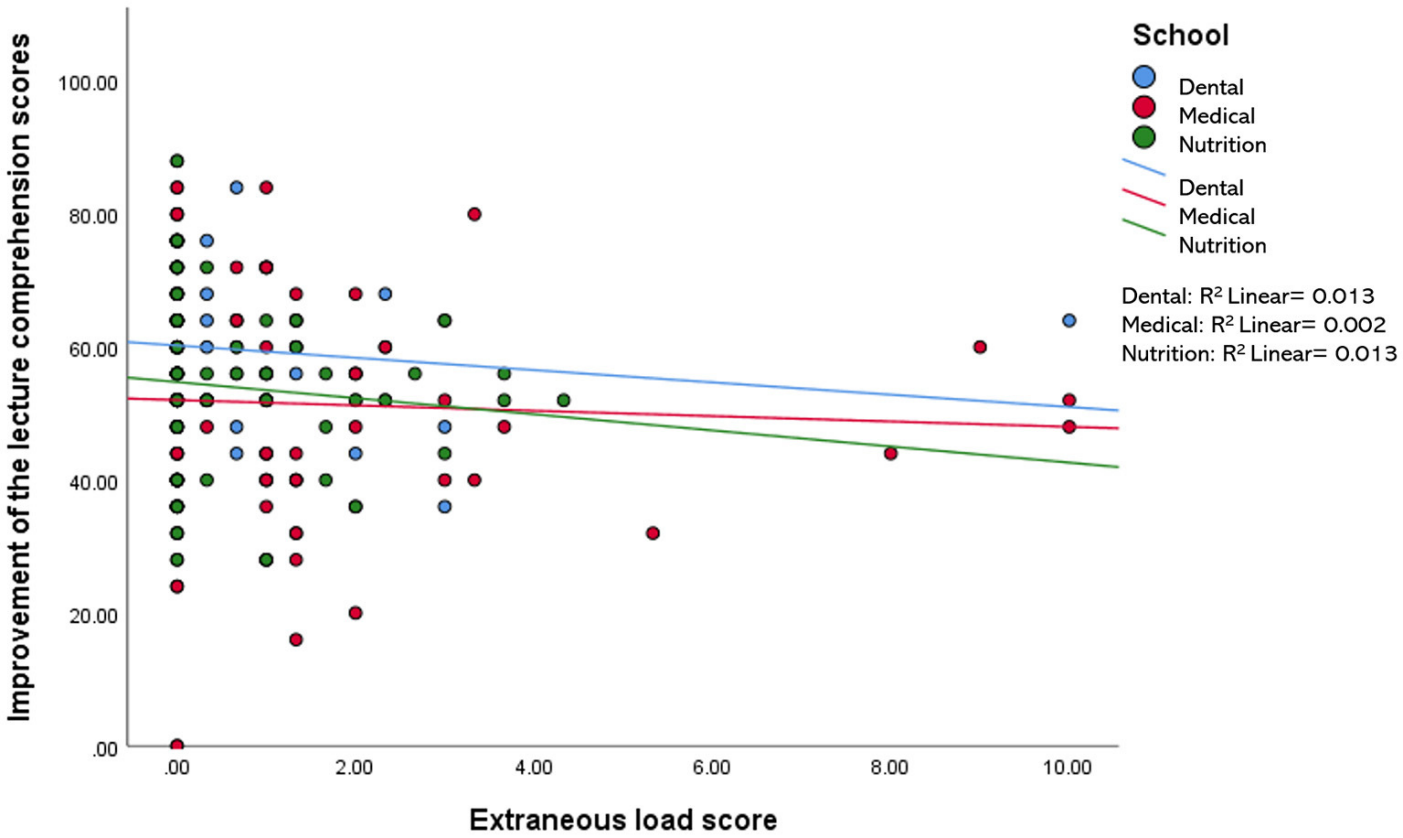
Improvement of the lecture comprehension scores vs. the extraneous load score after the CLT-based online lecture.

**Figure 4:**
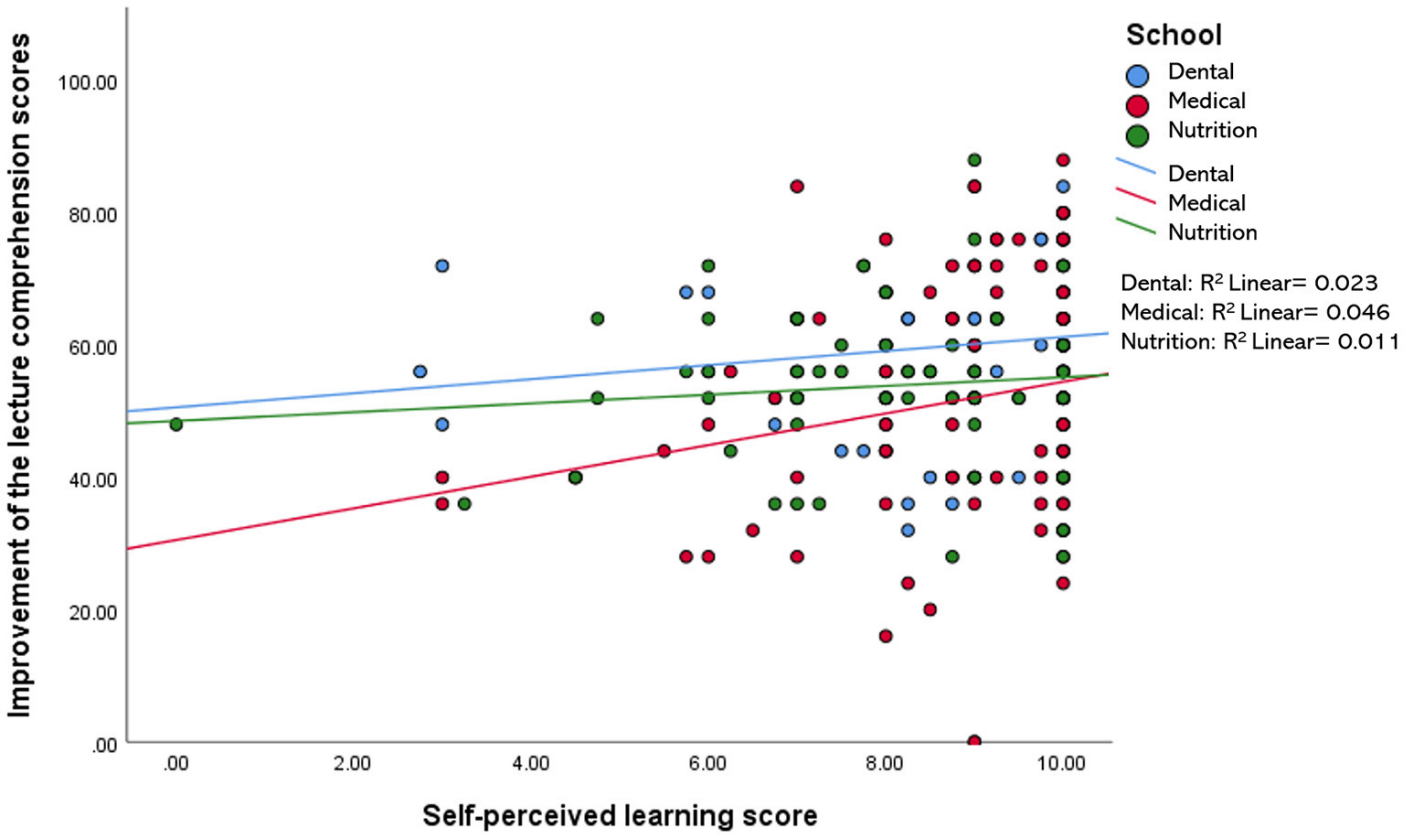
Improvement of the lecture comprehension scores vs. the self-perceived learning score after the CLT-based online lecture.

**Figure 5:**
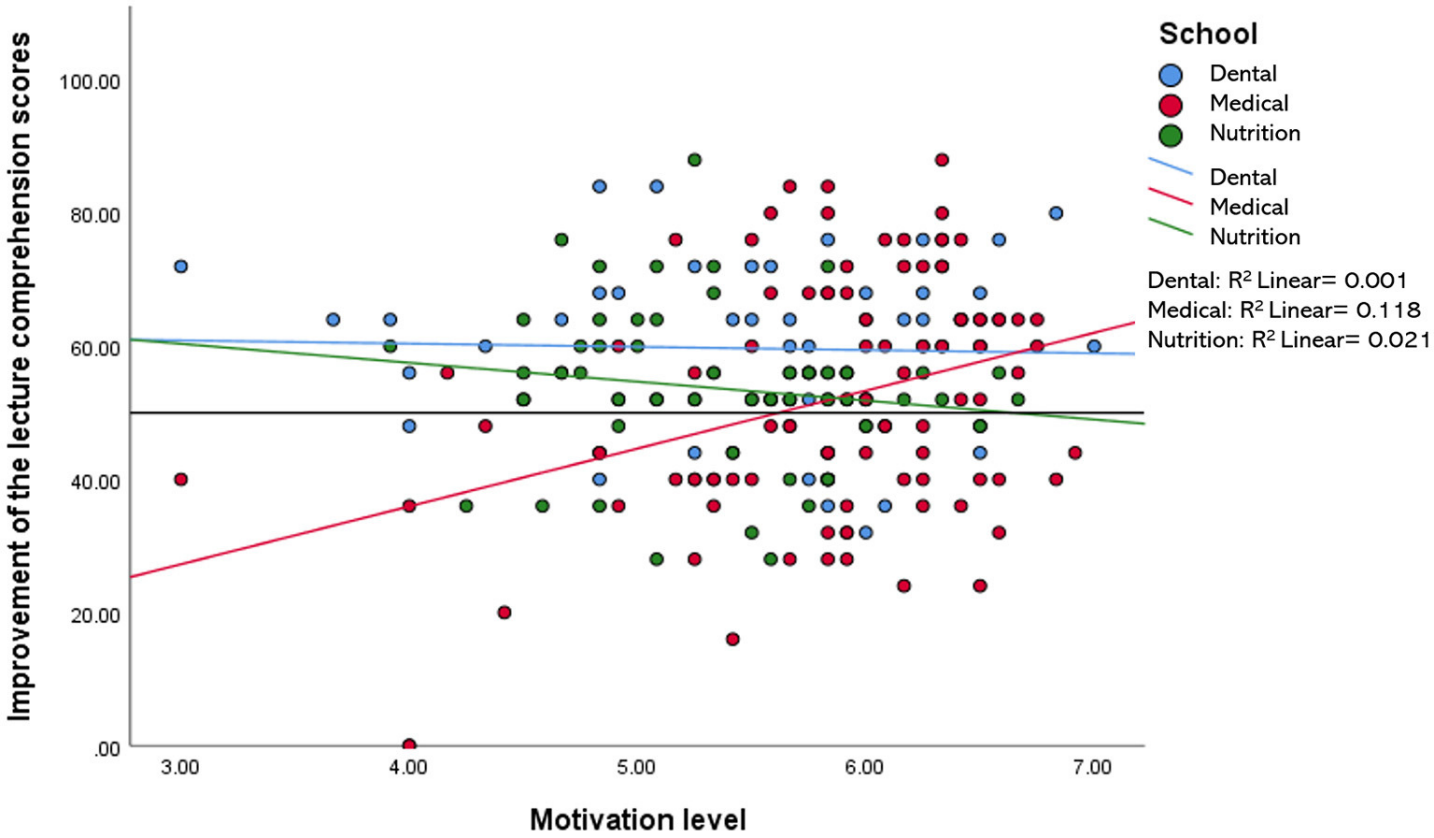
Improvement of the lecture comprehension scores vs. the students' motivation level after the CLT-based online lecture.

**Figure 6:**
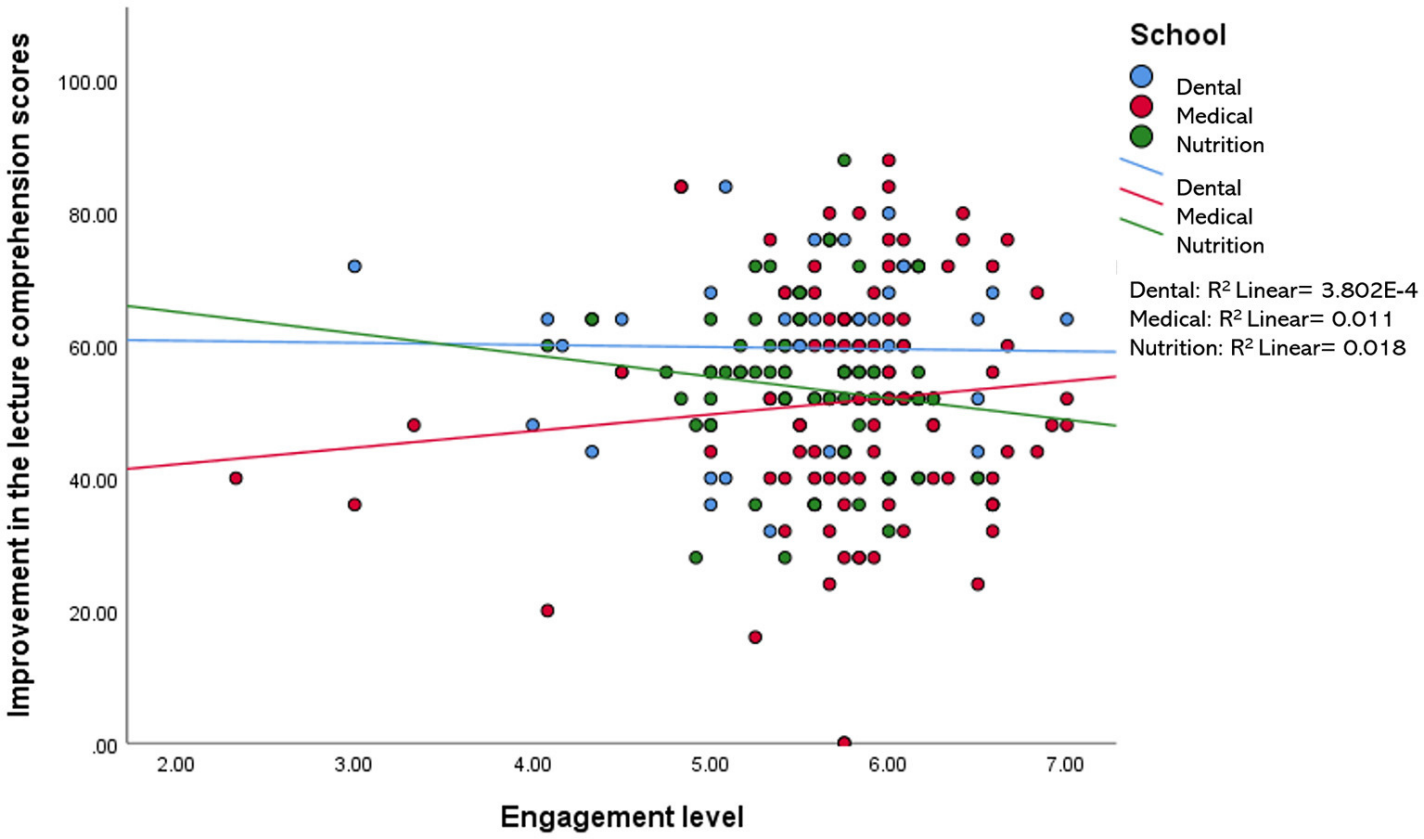
Improvement of the lecture comprehension scores vs. the students' engagement level after the CLT-based online lecture.

When compared among the three disciplines (i.e., medical, dental, and nutrition), the relationship between the improvement of the lecture comprehension scores and each of the variables measured in this study (i.e., motivation, engagement, IL, EL, and self-perceived learning scores) were found to be minimal, accounting for 0.2–11.8% of the variation in the data. For example, a statistically significant *R*^2^ value for the relationship between the improvement of the test scores and IL was only observed in the nutrition group−7.3% of the improvement in the test scores of the nutrition students may be explained by the reduction in their intrinsic load level (Figure 2). Likewise, the reduction in EL and the increment in self-perceived learning minimally influenced the improvement in the test scores of students in all three disciplines, with 0.2–1.3% variation in the data (Figures 3, 4). Similarly, improvements in the test scores were marginally influenced by the motivation and engagement levels following the CLT-based online lecture (Figures 5, 6). Nevertheless, the motivation level of the medical students seemed to influence the improvement in their test scores, with an *R*^2^ value of 0.118 accounting for 11.8% of the variation (Figure 5).

## Discussion

This study provided significant evidence regarding the effectiveness of CLT-based online lectures across different disciplines within the medical and allied health sciences programs. In general, the study proved that CLT-based online lectures promotes students' understanding of the lecture content, self-perceived learning, engagement toward the learning material, and motivation to learn. It was also found to be effective at reducing students' mental burden through the reduction of intrinsic and extraneous loads. Despite these findings, the CLT- based online lecture did not influence the students' engagement and motivation in the medical and nutrition disciplines. However, a within-groups comparison revealed that the variations in the data regarding the improvement in the lecture comprehension scores were marginally explained by the individual variables measured in this study. These findings indicate that the variations in the data regarding the improvement in the lecture comprehension scores could not be explained by individual factors; rather, this required a combination of the outcome variables to be working together during the learning process.

Online lectures are not a new concept in higher education. They were in pervasive use even before the outbreak of the COVID-19. The effectiveness of online lectures has been widely explored; however, the findings are inconsistent across different studies (15, 58, 59). A scoping review of online lectures in undergraduate medical programs reported improvements in students' learning outcomes when online lectures were used as an adjunct to normal face-to-face classes. However, the review identified cultural lag in adopting educational theories—particularly the multimedia learning theory—in online lecture design, which the authors believed could optimize students' learning achievements (60). Therefore, the present study makes several noteworthy contributions to the effectiveness of online lectures. First, this study adopted the empirically proven strategies of CLT and CTML into the design of online lectures. Second, it applied a synchronous approach to online lectures, which was comparable to the face-to-face live lecture.

The higher improvement in the post-lecture test scores after attending the CLT-based online lectures indicates that the students gained a better understanding of the lecture contents, which could have been influenced by the CLT effects. This result is in line with that of previous CLT research documenting improvements in students' performance from the pre- to post-intervention test scores following exposure to CLT-based instruction (61–63). Although it could be argued that students are often overloaded with information during a lecture, this study proved that a well-designed lecture can be easily intelligible to students, especially when it is designed to fit the human cognitive function. This finding supports the fact that CLT-based online instruction freed some of the students' working memory resources, which were then utilized to process the relevant subject content (64). Consequently, the processed information (i.e., schema) were transferred to the long-term memory for permanent storage, thereby indicating actual learning (65). When actual learning occurs, students are better able to understand the content of the instruction that they are learning (66).

Similarly, in this study, the improvement in the students' tests scores could indirectly indicate a reduction in the students' total cognitive load during the CLT-based online lecture. This was confirmed by the significant reduction in the students' IL and EL after they attended the CLT-based online lectures. These two types of cognitive loads contributed to the total cognitive load (26), and thus, managing these loads during instructional design and delivery was pertinent to the prevention of cognitive overload (i.e., total cognitive load exceeded the working memory resources) (67, 68). These findings are similar to those of a previous study by Hadie et al. (12) on the effectiveness of CLT-based lectures delivered face-to-face. Likewise, a study by Andrade et al. (69), revealed negative correlations between post-test scores and ECL scores in all students who were exposed to different multimedia instructions that were designed according to the CTML theory. When the teaching strategies and learning materials are designed effectively, it will increase the students' concentration and reduce the amounts of mental effort used to process the EL. Hence, more mental effort—which represent the GL—were directed for processing IL Andrade et al. (70). In light of the replicability of the results, the impact of CLT-based online lectures on students' cognitive load can be considered valid and reliable, at least in the context of various disciplines in the medical and allied health sciences.

Two possible postulations of successful IL reduction were the stimulation of prior knowledge and the systematic introduction of information during the lecture. At the beginning of each CLT-based online lecture, the students were given a pre-lecture task in the form of a quiz or video to stimulate or instill prior knowledge. It was reported that stimulation of prior knowledge would prevent unnecessary use of working memory resources in an attempt to retrieve prior knowledge (i.e., previously stored schema) from long-term memory (71). By helping the students retrieve their prior knowledge, their working memory resources were reserved and would suffice for information procession (71). Likewise, introducing information in a systematic manner could also reduce the intrinsic load, as explained by the isolated interacting element effects of CLT (72). In this research context, the lecturers were trained on how to apply the “chunking mechanism” concept, allowing them to gradually introduce information and combine this isolated information at a later stage of the lecture once the students had gained some understanding of the isolated components (73).

In this study, the reduction in EL could have been due to successful eliminations of redundant and irrelevant input during the lecture. This condition could have been reached through several lecturing strategies utilizing certain CLT effects related to EL (i.e., modality, split-attention, redundancy, and expertise reversal effects). For instance, the lecturers were guided on how to stimulate students' visual and auditory sensory modalities during the lecture through the application of slide preparation techniques and diagram management. Giving the facts that human working memory has both visual and auditory centers (i.e., a visuospatial sketchpad and phonological loop, respectively) (74), verbal explanations using one's own words and visual stimulus through systematic illustration were enforced during the CLT-based online lecture. This strategy ensured that the students optimize the use of their working memory capacity for information processing by utilizing both auditory and visual centers. Likewise, the use of other strategies (e.g., proper font size and color coding, appropriate animations and cues, and removal of redundant information) were aligned with other cognitive load effects aimed at preventing the unnecessary use of working memory resources in processing information that is irrelevant to learning (75, 76).

In addition, this study revealed significant increments of germane load, as represented by the increase in the self-perceived learning measure after the students had attended the CLT-based online lecture. These findings correspond with those of previous studies documenting increases in GL measures when students were exposed to CLT-based instructions (77–79). This finding supports the fact the reduction in EL is often accompanied by increases in GL load as more working memory resources were reserved for processing IL rather than EL (80). Although it may be argued that GL is not an independent construct—as it is assumed to be indistinguishable from IL (81, 82)—this study contributes to the body of evidence showing that GL moderates IL levels, as reported in several previous studies (83–85). Thus, GL is a reflection of students' efforts toward devoting their cognitive resources toward better comprehension of the lecture content and, thus, could be represented by the self-perceived learning measure.

GL requires a conscious mental effort or effortful strategies invested in learning. Previous studies have documented a direct association between GL and motivation, engagement, and the metacognition construct (86, 87). Similarly, this study found a significant increase in the motivation and engagement levels of the overall cohort of students who attended the CLT-based online lecture. These findings strengthen the indication that students possess a greater ability to invest their cognitive resources in information processing when they are motivated to learn or engage in learning materials and processes (83). In fact, the CLT-based lecturing strategies used in this study reinforced elements that can stimulate students' motivation and engagement (e.g., segmenting principle through the “pause and ask” strategy; personalization and voice principles through the provision of forethought, learning purpose, and reassurance; and self-explanation principle through clinical applied and mind-blowing quizzes). Moreover, it has been reported that the self-perceived learning construct is an important determinant for self-appraisals of academic competence as it influences actual learning capability, motivation, academic resiliency, persistence, and performance (88). Thus, we postulate that CLT-based online lectures successfully instill higher self-perceived learning through continuous encouragement and feedback from the lecturer as well as engaging learning experiences through the application of CLT and CTML principles.

In addition, this study demonstrated an intriguing and elusive relationship between improvements in the lecture comprehension scores in each of the variables measured: IL, EL, self-perceived learning, motivation, and engagement. Given the fact that each variable contributed marginally to the improvement in the lecture comprehension score, it could be argued that these variables could have interacted with each other during the learning process, thereby explaining the improvement in the test scores. For instance, EL might have been inextricably bound to GL elements (i.e., self-perceived learning, motivation, and engagement), as reported in a previous study (89). Furthermore, studies have shown that overall mental workload measures are modulated by attention, focus, alertness, prior learning experience, familiarity with learning tools, and the learning atmosphere (80, 90). Therefore, the current study makes an important contribution to the literature on online lectures by highlighting the importance of the optimal design and delivery of online lecture material that concurrently addresses all three components of cognitive load.

## Conclusion

Taken together, the findings of this research contribute in several ways to our understanding of the effectiveness of CLT-based lectures. First, the CLT-based lecture is applicable and feasible across different lecture delivery approaches, including face-to-face and online approaches. Second, CLT-based lectures are effective at promoting students' learning across different disciplines and at a high difficulty comprehension level, in the context of medical and allied health sciences. Third, the results of previous studies were replicated in this present study, thereby contributing to the validity and reliability of the CLT lecturing guideline. Indeed, this study has proven the continued relevance of the lecturing method for students' learning. The study also provided a resolution to the perceived limitations of the lecturing method, which is based on a solid empirically proven instructional design theory.

Nevertheless, this study was limited in several ways. The most important limitation lay in its study design—the pre-post quasi experimental design. The unprecedented COVID-19 pandemic and the implementation of the national lockdown in Malaysia hindered the accomplishment of the planned crossover design as face-to-face lectures were prohibited during the pandemic period. The disruption was integrated into the study to reflect the reality of student comprehension as it occurred in real life and we saw the possible benefits of CLT-based online lectures. To increase robustness, further studies are encouraged to have similar lesson settings as controls, e.g., online lesson with and without (controls) CLT-based applications. Furthermore, the generalizability of these findings are limited to the context of medical and allied health sciences programs. Thus, further research is needed to investigate the applicability and impact of CLT-based online lectures in non-medical disciplines. To achieve a better comparison of the results with minimal confounding factors, it is also recommended that future studies adopt randomized trials with a crossover design.

## Data Availability Statement

The raw data supporting the conclusions of this article will be made available by the authors, without undue reservation.

## Ethics Statement

The studies involving human participants were reviewed and approved by Human Research Ethics Committee, Universiti Sains Malaysia (USM/JEPeM/19070414). The patients/participants provided their written informed consent to participate in this study.

## Author Contributions

SH had a major role initiating the idea, constructing the conceptual framework of the study, managing the data collection session, and involved in manuscript writing. VS was involved in data analysis and manuscript writing. NO and NN were involved in data collection and manuscript writing. HL and KK were involved in verification of the results and manuscript writing. All authors contributed to the article and approved the submitted version.

## Funding

This work was supported by the Geran Penyelidikan Akademik Universiti Sains Malaysia (Grant Number: 1001.PPSP.8080007).

## Conflict of Interest

The authors declare that the research was conducted in the absence of any commercial or financial relationships that could be construed as a potential conflict of interest.

## Publisher's Note

All claims expressed in this article are solely those of the authors and do not necessarily represent those of their affiliated organizations, or those of the publisher, the editors and the reviewers. Any product that may be evaluated in this article, or claim that may be made by its manufacturer, is not guaranteed or endorsed by the publisher.
